# Network Neighbors of Drug Targets Contribute to Drug Side-Effect Similarity

**DOI:** 10.1371/journal.pone.0022187

**Published:** 2011-07-13

**Authors:** Lucas Brouwers, Murat Iskar, Georg Zeller, Vera van Noort, Peer Bork

**Affiliations:** 1 Structural and Computational Biology Unit, European Molecular Biology Laboratory, Heidelberg, Germany; 2 Max-Delbruck-Centre for Molecular Medicine, Berlin-Buch, Germany; Centro de Investigación Príncipe Felipe, Spain

## Abstract

In pharmacology, it is essential to identify the molecular mechanisms of drug action in order to understand adverse side effects. These adverse side effects have been used to infer whether two drugs share a target protein. However, side-effect similarity of drugs could also be caused by their target proteins being close in a molecular network, which as such could cause similar downstream effects. In this study, we investigated the proportion of side-effect similarities that is due to targets that are close in the network compared to shared drug targets. We found that only a minor fraction of side-effect similarities (5.8 %) are caused by drugs targeting proteins close in the network, compared to side-effect similarities caused by overlapping drug targets (64%). Moreover, these targets that cause similar side effects are more often in a linear part of the network, having two or less interactions, than drug targets in general. Based on the examples, we gained novel insight into the molecular mechanisms of side effects associated with several drug targets. Looking forward, such analyses will be extremely useful in the process of drug development to better understand adverse side effects.

## Introduction

As almost 30% of drug candidates fail in clinical stages of drug discovery due to toxicity or concerns about clinical safety [1], an increased understanding of unwanted side effects and drug action is desirable. Large-scale computational analyses of chemical and biological data have made it possible to construct drug-target networks that can be correlated to physiological responses and adverse effects of drugs and small molecules [2]. Such drug side effects have been predicted from the chemical structure of drugs [3], can be implied if drugs use a similar target or have been used themselves to predict new (off-)targets of drugs [2], [4], [5]. Even complete networks of pharmacological and genomic data have been used to identify drug targets[6].

Since most drugs have in addition to their primary target many off-targets [7], they are expected to perturb many metabolic and signaling pathways, eliciting both wanted and unwanted physiological responses. Such effects are expected to be part of a larger set of mechanisms that can explain the molecular basis of side effects, such as dosage effects, insufficient metabolization, aggregation or irreversible binding of off-targets [8]. To obtain a better understanding of the molecular mechanisms of disease, drug action and associated adverse effects, it makes sense to view chemicals and proteins in the context of a large interacting network [9], [10]. Integration with the drug-therapy network [11] and the analysis and intentional targeting of the protein interaction network underlying drug targets could expand our current range of drug treatments and reduce drug-induced toxicity [12], [13].

Previous integrative studies of human disease states, protein-protein interaction networks and expression data have uncovered common pathways and cellular processes that are dysregulated in human disease or upon drug treatment [14], [15]. However, the direct connection between the targeting of metabolic and signaling pathways by drugs and the adverse drug reactions that they cause has so far not been systematically studied and is only known for individual cases [16], [17], [18], [19], [20].

In this work, we aim to quantify the contribution of protein network neighborhood on the observed side-effect similarity of drugs. We developed a pathway neighborhood measure that assesses the closest distance of drug pairs based on their target proteins in the human protein-protein interaction network. We show that this measure is predictive of the side-effect similarity of drugs. By investigating the unique overlap between pathway neighborhood and side-effect similarity of drugs, we find known and unexpected associations between drugs and provide novel mechanistic insights in drug action and the phenotypic effects they cause.

## Results

### Network Neighborhood for predicting side-effect similarity

Our network neighborhood measure is based on the protein associations in the database STRING [21], which includes physical as well as functional and predicted interactions between proteins from human data as well as putative interactions transferred from other species. As there are large variations in number of interactions between proteins in STRING, we developed a normalized score based on the confidence-weighted edges in STRING, that reflects the closeness of drug targets in the protein-protein network (see Methods). The scores were normalized to find those associations between proteins that have significantly higher confidence score than the average confidence score of the edges of both proteins to all their network neighbors. We estimated the side-effect similarity of drug pairs using a previously described method ([4] and Methods, Table S1).

**Figure 1:**
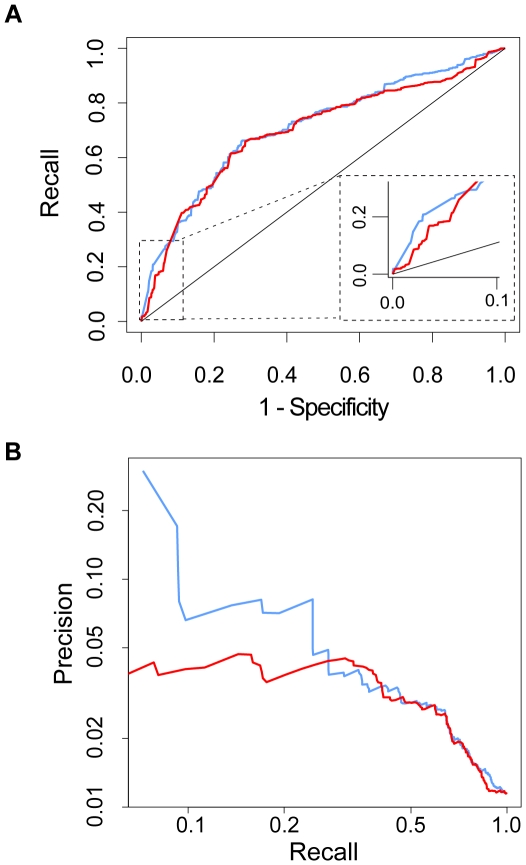
The predictive performance of normalized and direct pathway neighborhood scores for predicting side-effect similarity. This performance is estimated with a ROC curve (**A**) and a precision/recall plot (**B**). For these analyses, we discretize the side-effect similarity p-values into binary values at a cutoff of 0.10 as the target drug pairs to predict. This is a relatively strict cutoff that captures those drug pairs that are sufficiently similar in terms of their adverse effects. Blue: normalized pathway neighborhood scores Red: direct confidence scores STRING.

To investigate whether drug targets that are close to each other in the network tend to have similar side effects, both the normalized pathway neighborhood scores and the direct confidence scores in STRING were used to predict drug pairs with significant side-effect similarity (**Fig 1a**). As an overall correlation between interaction scores and side effect similarities cannot be found, we used ROC (Receiver Operating Characteristic) to address this question. The ROC curves show that both the normalized network measure and the direct confidence scores are able to predict drug pairs with side-effect similarity (P<0.01) with high recall and specificity. The area under curve (AUC) for the normalized pathway neighborhood is 0.71 and 0.70 for direct STRING confidence scores. The AUC increases for both measures if only drug pairs with higher side-effect similarity are considered (with AUCs of 0.76 and 0.75 at a cutoff of 0.01 for normalized and direct scores, respectively). The question arises whether drug target neighborhood could also be indicative of therapeutic effects, however we could not find such a relation (Figure S1).

Since both the Recall (7.9 %) of side-effect similarity by the top 500 normalized scores and the Precision (29.8%) are higher than the Recall and Precision by the top 500 direct neighborhood scores (1.8%; 5.7%) (see Methods/**Fig 1b**), the normalized pathway neighborhood scores indeed appear to be better suited for exploring the impact of pathway neighborhood on drugs causing similar adverse effects.

We conclude that drug pairs targeting proteins that are network neighbors indeed have higher side-effect similarity. However, while many drug pairs that have similar side effects target the same network neighborhood, protein network neighborhood doesn't appear to be a good predictor for novel, so far undetected side-effect similarities of drugs.

### Quantification of side-effect similarity caused by network neighborhood

Previous work has shown that sharing of drug targets is often reflected by similarity in side effects and now we find that also drugs targeting the same network neighborhood show similarity in side effects. We aim to quantify the percentage of side-effect similarities that arise from drugs that target a similar part of the protein-protein network as opposed to drugs that share a target. To this end, we define the drug pairs that target neighboring proteins as those that have a normalized neighborhood score ≥1, i.e. those protein pairs that have STRING confidence which are more than twice the average confidence of the proteins. At this cutoff, 25,263 drug pairs are classified as targeting the same protein network neighborhood.

**Figure 2:**
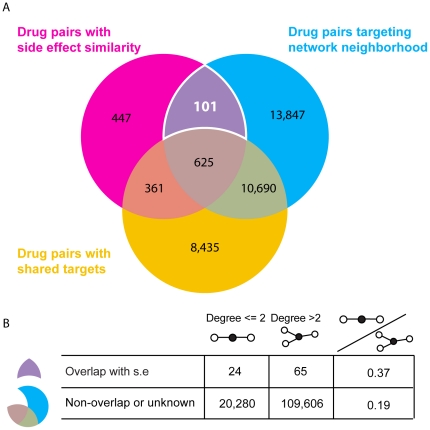
Drug pairs with side effect similarity overlap with drug drug pairs targeting network neighborhood. (A) Venn diagram of drug pairs with side-effect similarity, shared targets and targeting network neighborhood. We define drug pairs that have side effect p-values ≤0.10 as pairs having significant side-effect similarity. Pairs that target neighboring proteins are defined as having normalized neighborhood score ≥1. Drug pairs that share one or more drug targets are based on data from DrugBank, Matador and PDSP Ki. Only drug-pairs are taken into consideration where at least one drug target is known for both drugs and the side-effect similarity is also available. After removing 12 drug pairs (from 101) where we might expect target-sharing based on chemical or protein similarity, 89 drug pairs are left that target neighboring proteins and have similar side-effects. This is 5.8% of drug pairs with side-effect similarity where we have both target and network information. A minimum of 986 (64%) of side-effect similarities can be explained by sharing drug-targets in the set where at least one drug target is known. (B) Degree distribution of drug pairs with side-effect similarity that target the same network neighborhood. The drugs have been divided in two categories, drugs that target proteins with two or less interaction partners and more than two interaction partners. The drugs in drug pairs that have side-effect similarity target significantly more target proteins with fewer interaction partners than when we consider all drug pairs that target the same network neighborhood. Drug pairs with high chemical similarity or with high sequence similarity of protein binding partners have been removed from the overlapping set, to avoid possible undetected shared targets between drug pairs.

Of all drug pairs with significant side-effect similarity (N = 1,534), we observe that both drugs are targeting a similar protein network neighborhood in 47.3% of the cases (N = 726) (**Fig 2a**). However many of these similarities are expected to arise because drugs have one or more drug targets in common [4]. If we exclude those drug pairs that are known to share a target, the overlap is reduced to only 101 drug pairs with significant side-effect similarity.

Since it is known that drugs that are chemically similar or have targets that are similar in sequence and/or structure are likely to share a target [2], [22], we further exclude drug pairs that display chemical structure similarity and/or the sequence similarity of their targets. For chemical similarity, we consider Tanimoto coefficients ≥0.8 as structurally similar. Below this cutoff, less than 30% of these drug pairs are expected to have similar protein binding properties [23]. Four of the 101 drug pairs have similar chemical structures, reducing the overlap to 97 drug pairs. These 97 drug pairs have average Tanimoto coefficients of 0.28±0.18, showing that they are chemically unrelated and unlikely to share the same protein targets on these grounds. Of these 97 pairs, 9 drug pairs had protein targets that displayed sequence similarity (≤1e^−4^ using the BLAST algorithm [24]), resulting in a unique set of 89 (5.8%) drug pairs with side-effect similarity correlated with network neighborhood, while we estimate that at least 64% of significant side-effect similarities are explained by shared drug targets.

To investigate if the local network topology is markedly different for the proteins that are targeted by these drugs, we investigated the degree (number of interaction partners) of drug targets (**Fig 2b**). We find that these drugs significantly target proteins with low degrees, defined as an average degree of both targets ≤2 (p-value <0.0032). We only consider edges with STRING confidence scores >0.7, a cutoff that has previously been shown to capture relevant protein-protein interactions and functional pathway modules [25]. Thus, two proteins in a linear part of the network that are targeted by drugs are more likely to display similar side effects than two hub-like proteins with many interactions. We conclude that if either component in a linear pathway is targeted, similar molecular and physiological effects unfold.

### Exploration of network neighborhoods that influence side effects: Novel mechanistic insights of drug actions

**Figure 3:**
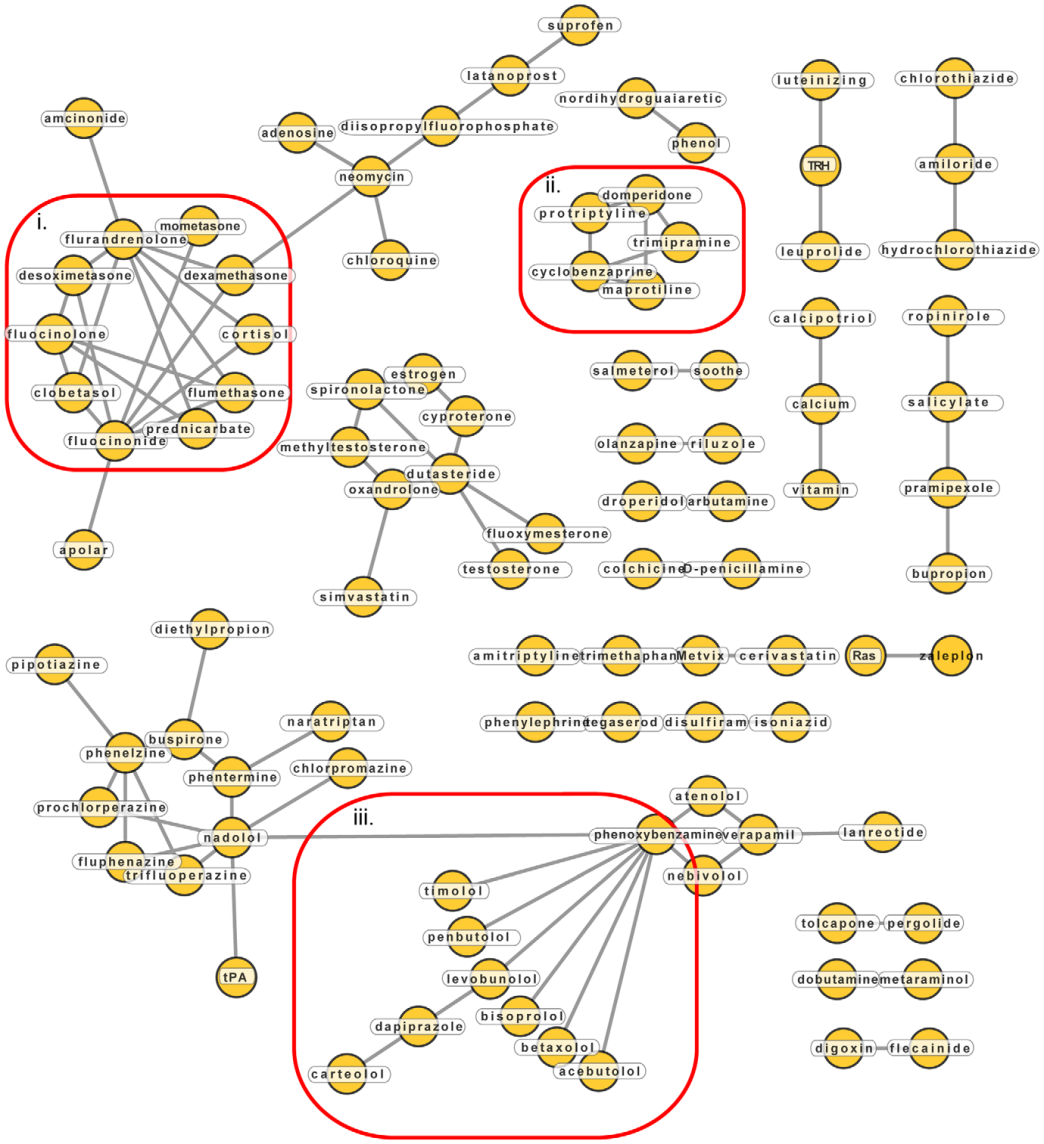
Drug-drug network of drugs targeting network neighbors and having side-effect similarity. Drugs are drawn as yellow circles, grey lines between them indicate drug targets that are network neighbors.

We visualized the drug-drug relationships of the 89 remaining cases in a network (**Fig 3**). In addition to many single edges between isolated drugs, a number of highly connected nodes can be seen. For example, the cluster of glucocorticoids (**i**), tryciclic antidepressants (**ii**) and α/β blockers (**iii**). While these drugs are chemically dissimilar and their targets have little sequence similarity, they have similar targets and mode of action, making their observed side-effect similarity relatively unsurprising.

The network analysis also reveals novel mechanistic insights, illustrated, for example by the association of the alcohol sensitivity drug disulfiram with isoniazid, which is both an antitubercular agent and antidepressant. Common adverse effects of both drugs include liver related pathologies (“jaundice”, “hepatitis”), but also neural and neuronal conditions (“encephalopathy”, “neuritis”, “psychosis”, “eye pain”). Both drugs have long been suspected to interact with each other when taken concurrently [26], presumably since they both interact with cytochrome P450 2E1 [27], [28]. However, our network neighborhood analysis reveals that both drugs also interfere with butanoate metabolism. Isoniazid is known to induce pyridoxine deficiency, affecting the breakdown of the major neurotransmitter GABA, since both GABA transaminase and glutamic acid decarboxylase require pyridoxine as a cofactor [29]. Disulfiram inhibits GABA transaminase and succinate-semialdehyde dehydrogenase, which are both involved in the catabolism of GABA [30]. These interactions might be responsible for the similarity of neural and neuronal side effects observed in patients taking these drugs.

Another example for revealing mechanistic insights of drug actions can be derived from the association between tegaserod and phenylephrine, both GPCR agonists. Tegaserod is an agonist of the serotonin receptor 5-hydroxytryptamine 4 (5-HT4) and has been used for treating chronic constipation in patients with irritable bowel syndrome and chronic idiopathic constipation [31], [32]. Tegaserod was withdrawn from the market when pooled clinical studies indicated an increased risk of cardiovascular ischemic events, even though a recent cohort study found no such association [33]. Phenylephrine is a beta-2 adrenergic receptor (ADRB2) agonist and its vasoconstrictive properties have been found useful in a wide range of applications, including use as a decongestant, vasopressor and pupil dilation agent [34]. The adverse effect profiles of both drugs are similar and include side effects of a cardiovascular (“angina pectoris”, “tachycardia”), neuronal (“dizziness”, “sleep disorders”) and genitourinary nature. Interestingly, some of these adverse effects appear to be opposite physiological responses (“somnolence” and “insomnia,” or “polyuria” and “dysuria” for tegaserod and phenylephrine, respectively). The 5-HT4 and ADRB2 proteins directly interact with each other in heterodimers and are therefore network neighbors [35]. The functional relevance of this dimerization is as of yet unknown, but it is tempting to speculate that the similar physiological effects of both drugs, including the cardiovascular adverse effects of tegaserod, have a common molecular basis in their functional interaction.

A final example is the association between the drugs tolcapone and pergolide, which are both used in the treatment of Parkinson's disease [36]. Both drugs have broad side effect profiles, and share many severe adverse effects of the nervous (“hallucinations”, “amnesia”), digestive (“gastroenteritis”, “diarrhea”) and cardiovascular (“bradykardia”, “stroke”) systems. Despite the large similarities in the physiological response of the human body, both drugs have different mechanisms of action: tolcapone is a catechyl-O-methyl transferase (COMT) inhibitor [37], whereas pergolide is a dopamine receptor agonist [38]. By inhibiting COMT, tolcapone increases dopamine levels by preventing it from being converted to 3-methoxytiramine. Additionally, tolcapone is often used in adjunct with the dopamine precursor levodopa, to reduce its rapid catabolization in the gut, thereby prolonging the effects of levodopa. As a dopamine receptor agonist, pergolide mimics the activating effects of dopamine on the dopaminergic receptors. Our analysis suggests that the observed similarity of side effects of both drugs might reflect the underlying physiological response to prolonged/increased dopaminergic activity.

## Discussion

In this study we have shown that the similarity of adverse effects for a number of drugs can uniquely be explained by the common protein subnetwork that they target. While network neighborhood on its own is not predictive for side-effect similarity, it does lead to novel mechanistic insights into the molecular basis of side effects. It must be noted that the percentage of drug pairs with significant side-effect similarity sharing a common target is much larger than the percentage of drug pairs targeting non-overlapping proteins that are neighbors in a pathway (64% compared to 5.8%). Previous studies relied on the assumption that common adverse effects between drugs generally arise due to the binding of the same (off-)targets [2], [4], [16], [19]. This seems to be a valid assumption since only a small number of side-effect similarities are expected to arise due to pathway neighborhood effects based on the results presented here.

The figure of 5.8% should be treated with caution and is likely to be an underestimate of the role of the protein interactions play in causing adverse drug effects. Since our pathway neighborhood measure only accounts for direct neighbors in the network, further relations between protein network neighborhood and phenotypic effects might be found if larger parts of the network are considered. The number is even more likely to increase if the limited knowledge of the human protein-protein interaction network, even after transferring information from other species, will be extended by more experimental data. The integration of protein network data with other molecular and cellular readouts (e.g., gene expression) should also provide a more sensitive and comprehensive understanding of the role that pathway perturbations play in establishing adverse drug reactions. On the other hand, more complete knowledge of the drug target profiles of small molecules could increase the number of side-effect similarities that are associated with a shared drug target, making our figure an overestimation.

In the drug-drug network that is presented here, we observe multiple drug pairs where both drugs are known to negatively interact (such as disulfiram and isoniazid) or are used in combination therapies (amiloride and thiazide, for example). Most *in silico* predictions of adverse drug interactions are currently based on either cytochrome P450 metabolization information or pharmocokinetic predictions derived from *in vitro* or *in vivo* data [39], [40]. The protein-protein network has so far remained underexplored in the prediction of adverse drug interactions [12], [16], [17], [18], [19], [20]. With the expansion of human protein-protein interaction networks and pathway information, neighborhood analysis as is presented here can be refined and adapted for the prediction adverse drug interactions or efficacious drug combinations.

## Materials and Methods

### Construction of drug target, side effect and pathway effect datasets

Drugs and their protein targets were extracted from the drug target databases; DrugBank, Matador [41], [42] and PDSP K_i_ (http://pdsp.med.unc.edu/indexR.html). Only drug target annotations with binding constants lower than 10 µM were considered. Metabolizing enzymes and proteins like albumin that bind drugs unspecifically were excluded from the drug target set because our goal in this research is to identify shared side effects of drugs that target functionally related proteins on a level that extends beyond basic interactions. Proteins were excluded if their ENSEMBL annotations matched one of the following keywords: “Cytochrome”, “ATP-binding cassette”, “Thromboxane”, “Arachidonate-lipoxygenase”, “Glutathione-transferase”, “Flavin containing monooxygenase”, “Albumin” or “Histocompatibility”. The resulting drug target set is composed of 781 drugs and 1245 targets, forming 6036 drug target interactions.

In order to investigate the role of pathways in the side-effect similarity of drugs, we created two datasets: one for pairwise comparisons between drugs in terms of the adverse effects that they cause and another one that contains a measure for the closeness of proteins in the human protein-protein network. The side-effect similarity of drug pairs is calculated as previously described [4]. In short, side effects are extracted from publicly available package inserts via text-mining approaches. To capture the similarity between closely related side effects, side effects are mapped to the Unified Medical Language System ontology after which all parent terms are assigned to the drugs. For every drug pair a side-effect similarity score is calculated based on the side effects that they share, where every shared side effect is weighted for the rareness and its correlation with other side effects. Drugs and their side effects are available for download from SIDER (http://sideeffects.embl.de) [43].

To obtain a measure for the relatedness of proteins in the human protein-protein network, we use the confidence scores between proteins in the STRING functional protein association database [21]. STRING is a resource that not only captures physical protein-protein interactions, but also functional associations derived from multiple sources, such as manually curated pathway databases and inferred relationships from text-mining PubMed abstracts. Since drugs may cause similar side effects on different functional levels, ranging from pathway perturbations to targeting the same protein complexes, this integrative approach suits our goals by allowing us to analyze the relationships between proteins on several molecular and functional levels at once.

For every possible pair of drugs in our dataset of 827 drugs, we go through the list of their associated targets and retrieve the confidence scores for every target pair where there is an edge present in the STRING network. We normalize these confidence scores by dividing them by the sum of the average confidence scores of all edges both targets have in the network. The idea behind this normalization is that an interaction with high confidence between two proteins is more significant if it has a higher confidence score than would be expected from the average confidence score of the edges of both proteins. The overlap between the datasets on side-effect similarity and pathway neighborhood consists of 129,975 unique drug pairs.

### Chemical similarity of drugs

The chemical similarity of drugs is calculated using the commonly used Tanimoto/Jaccard 2D chemical similarity scores [44]. The structural resemblance between two molecules is calculated by dividing the intersection of chemical substructures common to the pair of molecules by the total number of chemical substructures found in both pairs.

### Normalization

For every possible pair of drugs in our dataset of 827 drugs, we go through the list of their associated targets and retrieve the confidence scores for every target pair where there is an edge present in the STRING network. We normalize these confidence scores by dividing them by the sum of the average confidence scores of all edges 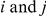
that both targets u_1_ and u_2_ have in the network, according to equation 1. The highest normalized score of a target pair is then reported as the normalized pathway neighborhood score for a drug pair.
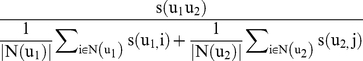
(1)


The idea behind this normalization is that an interaction with high confidence between two proteins is more significant if it has a higher confidence score than would be expected from the average confidence score of the edges of both proteins.

## Supporting Information

Figure S1 **The predictive performance of normalized and direct pathway neighborhood scores for predicting therapeutic effect similarity.** This performance is estimated with a ROC curve (**A**) and a precision/recall plot (**B**). For these analyses, we take as positive set drugs that overlap at the 3^rd^ level of ATC classification and as negative set all other combinations of these drugs. Although there is some signal, there seems to be no significant overlap between drug target neighborhood and drug therapeutic effect similarity.
(TIF)

Table S1 **Side effect similarity between drugs.**
(XLSX)
