# Supplementary figures and images for: Network Neighbors of Drug Targets Contribute to Drug Side-Effect Similarity
Source: PLoS One. 2011 Jul 13;6(7):e22187. doi: 10.1371/journal.pone.0022187 (PMC3135612; doi:10.1371/journal.pone.0022187)

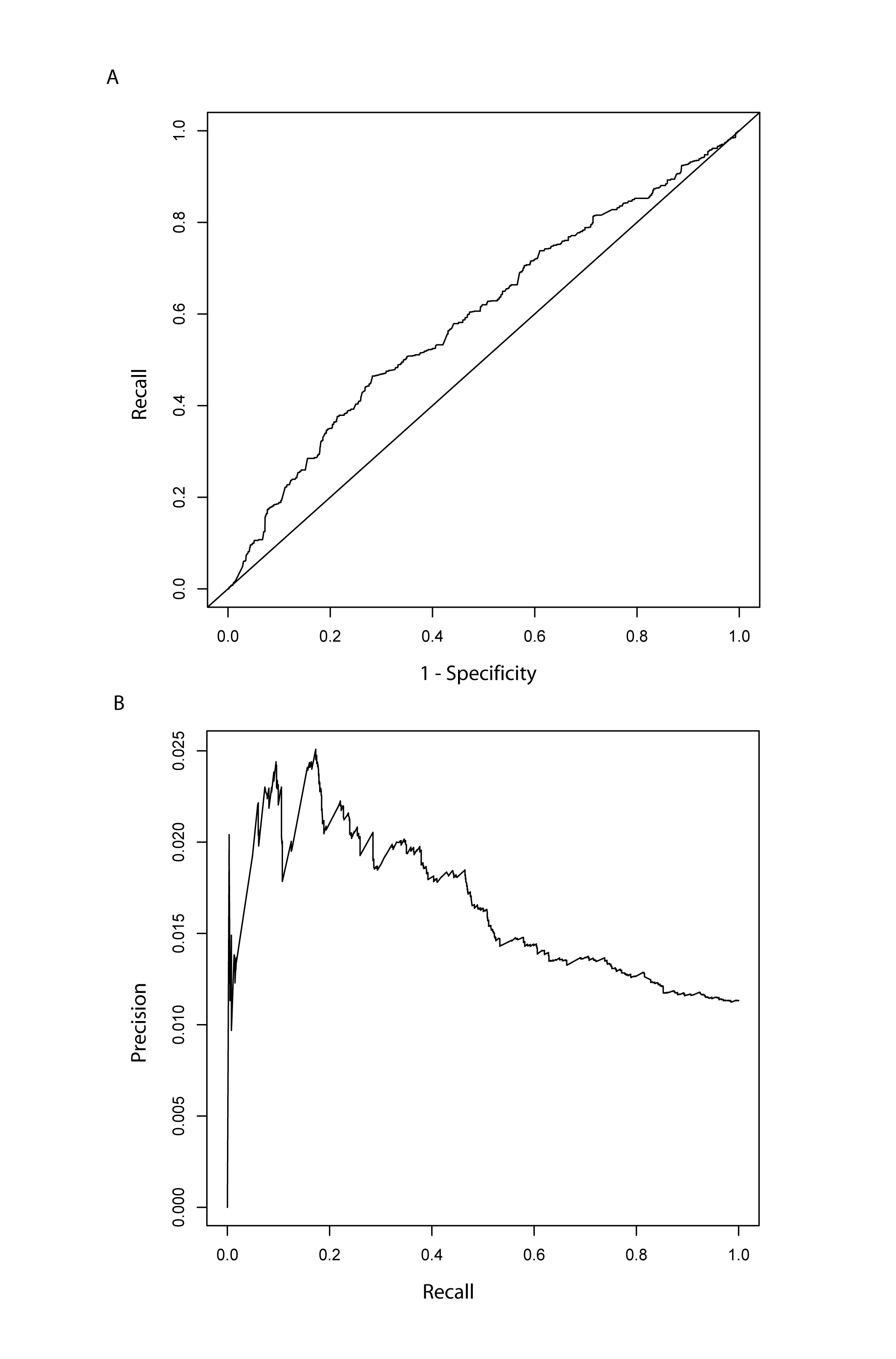

Supplement: Figure S1 — The predictive performance of normalized and direct pathway neighborhood scores for predicting therapeutic effect similarity. This performance is estimated with a ROC curve (A) and a precision/recall plot (B). For these analyses, we take as positive set drugs that overlap at the 3rd level of ATC classification and as negative set all other combinations of these drugs. Although there is some signal, there seems to be no significant overlap between drug target neighborhood and drug therapeutic effect similarity. (TIF) [file pone.0022187.s001.tif]
